# Supervised Learning and Large Language Model Benchmarks on Mental Health Datasets: Cognitive Distortions and Suicidal Risks in Chinese Social Media

**DOI:** 10.3390/bioengineering12080882

**Published:** 2025-08-19

**Authors:** Hongzhi Qi, Guanghui Fu, Jianqiang Li, Changwei Song, Wei Zhai, Dan Luo, Shuo Liu, Yijing Yu, Bingxiang Yang, Qing Zhao

**Affiliations:** 1 College of Computer Science, Beijing University of Technology, Beijing 100124, China; qhz123@emails.bjut.edu.cn (H.Q.);; 2 Institut du Cerveau—Paris Brain Institute-ICM, Sorbonne Université, CNRS, Inria, Inserm, AP-HP, Hôpital de la Pitié-Salpêtrière, 75013 Paris, France; 3 School of Nursing, Wuhan University, Wuhan 430071, China

**Keywords:** mental health, social media, cognitive distortions, suicide detection, large language model, deep learning

## Abstract

On social media, users often express their personal feelings, which may exhibit cognitive distortions or even suicidal tendencies on certain specific topics. Early recognition of these signs is critical for effective psychological intervention. In this paper, we introduce two novel datasets from Chinese social media: SOS-HL-1K for suicidal risk classification, which contains 1249 posts, and SocialCD-3K, a multi-label classification dataset for cognitive distortion detection that contains 3407 posts. We conduct a comprehensive evaluation using two supervised learning methods and eight large language models (LLMs) on the proposed datasets. From the prompt engineering perspective, we experiment with two types of prompt strategies, including four zero-shot and five few-shot strategies. We also evaluate the performance of the LLMs after fine-tuning on the proposed tasks. Experimental results show a significant performance gap between prompted LLMs and supervised learning. Our best supervised model achieves strong results, with an F1-score of 82.76% for the high-risk class in the suicide task and a micro-averaged F1-score of 76.10% for the cognitive distortion task. Without fine-tuning, the best-performing LLM lags by 6.95 percentage points in the suicide task and a more pronounced 31.53 points in the cognitive distortion task. Fine-tuning substantially narrows this performance gap to 4.31% and 3.14% for the respective tasks. While this research highlights the potential of LLMs in psychological contexts, it also shows that supervised learning remains necessary for more challenging tasks.

## 1. Introduction

The prevalence of mental illness, particularly depression, continues to present significant challenges worldwide. According to a 2023 report from the World Health Organization (WHO), an estimated 3.8% of the global population experiences depression [1]. Specifically in China, mental health challenges are equally severe. The results of a landmark nationwide study, the China Mental Health Survey (CMHS), published by Huang et al. (2019) [2], revealed that the weighted lifetime prevalence of depressive disorders among Chinese adults is 6.8%, highlighting the pervasiveness of these mental health issues in the country. Such severe depression can often precipitate suicidal behaviors [3]. Platforms such as Twitter and Sina Weibo have become channels for emotional outbursts as social media has become more widespread [4]. Within these platforms, a certain subset of topics recur, with users frequently expressing deep-seated negative emotions and even showing suicidal tendencies [5,6].

Artificial intelligence (AI), especially the branches of deep learning and natural language processing, is a promising solution to this challenge [7]. Over recent years, AI research has resulted in the formulation of several algorithms tailored for emotion recognition within textual data [8]. However, traditional sentiment analysis, which primarily categorizes text as positive, negative, or neutral, is often insufficient. It fails to capture the underlying maladaptive reasoning patterns that define cognitive distortions, which often involve complex patterns of irrational thinking beyond surface sentiment and are more indicative of mental health risks. Moreover, these advancements are not without obstacles [9]. Constructing a potent deep learning model often demands considerable time and financial resources. The cost of data labeling, the need to engage domain experts, and the variability of model performance when moved to different application domains present significant challenges [10]. In specialized domains such as mental health, annotation must be performed by trained clinical professionals rather than crowd workers. For example, in a study on cognitive reframing, Sharma et al. [11] recruited 15 practicing mental health professionals and clinical psychology graduate students to label data, paying them CNY 37.50 per hour. This significantly exceeds the typical cost of annotation on crowdsourcing platforms, making it financially burdensome to scale high-quality datasets. This highlights a critical need for more flexible and adaptable algorithmic solutions, especially in the mental health domain [12]. In this context, the emergence of large language models (LLMs) is particularly noteworthy, and they have already attracted significant attention in the mental health domain [13].

Characterized by their large parameter and training datasets, LLMs stand as the state of the art in the framework of computational linguistics [14,15]. Their potential lies in their ability to comprehend and emulate human-like text nuances. Despite their promising potential, several studies have attempted to validate their practical performance. For instance, Xu et al. [16] examined four public datasets related to online social media sentiment detection. However, their study focused only on English data, and the classification task was relatively broad. This reveals a critical gap in Chinese-language mental health research, where cultural and linguistic nuances, such as indirect expressions, euphemisms, and idiomatic usage, affect how psychological distress is conveyed. These differences underscore the necessity of Chinese-specific datasets and highlight the novelty of our work on Chinese social media. A nuanced understanding of emotional expression is particularly important for the early detection of mental health issues, enabling timely and accurate intervention. Such capabilities further emphasize the importance of fine-grained emotion recognition in psychological assessment and therapy. Nevertheless, due to the lack of large-scale evaluations and real-world testing, concerns remain about the reliability of LLMs in high-stakes domains such as medicine and healthcare [15].

However, so far, there is a lack of large-scale datasets for psychology-related tasks, especially cognitive distortions. Sharma et al. [17] proposed an English dataset consisting of 600 entries with 15 labels, 14 of which pertain to cognitive distortions, making it a multi-label classification dataset. Despite the diversity of cognitive distortion labels, the dataset’s sample size is relatively small. The dataset, named Therapist Q&A, is available at https://www.kaggle.com/arnmaud/therapist-qa (accessed on 22 May 2025). It includes 2507 entries with 11 labels, 10 of which are cognitive distortion labels, also categorized as a multi-label classification dataset. In contrast, the landscape for Chinese datasets has been particularly constrained, not only in scale but also in annotation complexity. To the best of our knowledge, one of the first public resources is the C2D2 dataset [18]. However, to save costs, its authors abandoned the multi-label annotation approach in favor of single-labeling. This trend of simplification is further evidenced in the latest Chinese dataset by Lin et al. [19], which circumvents the challenge of classification altogether by focusing only on the binary detection of whether a distortion exists. In practice, it is common for a single text to reflect multiple cognitive distortions simultaneously. Our work is constructed with a label scheme methodologically distinct from existing works. We directly adopt the classic framework from Burns [20], a foundational standard in Cognitive-Behavioral Therapy (CBT), whereas other datasets often utilize inconsistent labeling systems. Consequently, our work represents the first systematic application of this authoritative clinical framework to the task of Chinese multi-label classification, ensuring our dataset’s professional quality and practical utility.

Despite increasing attention to suicide risk detection, data accessibility remains a major challenge. For example, the CLPsych 2024 shared task utilized the dataset to identify evidence of suicidality in Reddit posts, but the dataset was not publicly available due to privacy concerns [21]. The CLPsych 2025 task similarly adopted a password-protected dataset requiring formal agreements [22]. These restrictions underscore the broader difficulty of accessing high-quality annotated resources, even in English. In response, some efforts have aimed to improve openness. The RSD-15K dataset [23], for instance, provides around 15,000 Reddit posts with four-level suicide risk annotations and is publicly available upon request. However, such resources remain largely English-centric. In contrast, the lack of publicly available datasets in Chinese is even more severe. Cao et al. [24] created a binary classification dataset based on Sina Weibo, though not all posts reflect explicit suicidal intent. Huang et al. [25] collected data from 130 individuals who died by suicide, but the dataset is no longer accessible. These limitations highlight the urgent need for high-quality Chinese-language resources to advance suicide detection research in non-English contexts.

To provide more contribution to the research community, we present two datasets: SOS-HL-1K for high and low suicide risk classification, and SocialCD-3K for a cognitive distortion task. The SOS-HL-1K dataset contains 1249 social media posts reflecting high and low suicide risk, and the SocialCD-3K dataset contains 3407 social media posts annotated in a multi-label way. Both datasets were annotated by multiple domain experts. LLMs are prompt-driven and characterized by their flexibility and ease of use across different tasks. However, their specific performance in psychological tasks, especially in the Chinese domain, has not been fully explored. To address this, we evaluate the performance of eight LLMs and test the performance of four zero-shot and five few-shot prompt strategies on the two proposed datasets. We also test the performance of different types of LLMs fine-tuned for these tasks. The experimental results show a significant performance gap between prompted LLMs and supervised learning. This gap is 6.95 percentage points based on the F1-score for the high-risk class in the suicide task, widening to a more pronounced 31.53 points on the micro-averaged F1-score for the cognitive distortion task. Fine-tuning substantially narrows these gaps to 4.31% and 3.14%, respectively, though supervised learning retains the lead. To the best of our knowledge, our proposed dataset is the first Chinese cognitive distortion multi-label classification dataset. We evaluate the performance of LLMs from different angles, providing a valuable reference for researchers using LLMs in psychological tasks.

In summary, this work makes the following primary contributions:

1. We introduce SocialCD-3K, the first Chinese multi-label cognitive distortion dataset, constructed from Burns’ classic Cognitive-Behavioral Therapy (CBT) framework, unlike prior simplified or inconsistent schemes.
2. We present SOS-HL-1K, a publicly available Chinese social media dataset for suicide risk classification, addressing the scarcity of non-English resources.
3. We provide a benchmark evaluating two supervised methods and eight LLMs across zero-shot, few-shot, and fine-tuning settings, offering a reference for future research.
4. We analyze performance gaps between supervised methods and LLMs on simple vs. complex psychological tasks, revealing the current limitations of LLMs in specialized domains.

## 2. Related Work

### 2.1. Text Sentiment Analysis

Social media platforms have become central channels for the expression of emotions worldwide. Accurately and quickly identifying the emotions embedded in this data presents a big challenge to computational algorithms [8]. Fu et al. [26] developed a distant supervision approach for classifying high and low suicide risk on Chinese social media platforms, using annotations by experts at different levels, significantly reducing the dependency on extensive manual effort by professional experts. Employing this approach, coupled with the integration of key psychological features extracted from user blogs, they achieved an F1-score of 77.98%. Singh et al. [27] focused on COVID-19-related sentiment analysis on social media platforms, training a BERT-based model on these two datasets, achieving an accuracy of 94%. Wan [28] introduced a method for sentiment analysis of Sina Weibo comments, leveraging deep neural networks. The data undergo feature extraction through multi-level pooling and convolution layers, and key features are subsequently extracted from the feature matrix using a CNN. For the final classification and sentiment analysis, the softmax logistic regression method is employed. Zhang et al. [29] introduced a factor graph-based emotion recognition model that integrates correlations among emotion labels, social interactions, and temporal patterns into a unified framework, adeptly identifying multiple emotions through a multi-label learning approach.

Although deep learning algorithms demonstrate impressive performance, the requirement for significant volume of labeled data makes them difficult to apply. The utilization of the distant supervision approach proposed by Fu et al. [26] reduces the need for labeling but still involves expert groups. Given these limitations, there is a growing demand for efficient methods for emotion detection on social media. Recent advancements in LLMs offer potential solutions, attracting increased attention in the mental health domain.

### 2.2. Large Language Model and Its Applications in Medical Domain

The advent of Large Language Models (LLMs) like OpenAI’s GPT-4 has revolutionized natural language processing [30], significantly outperforming smaller models [31]. These models have diverse applications, including content generation [32], medical report assistance [33], coding assistance [34], education [35], and answering medical-related questions [36]. The large size of the model enables them to generate complex, contextually relevant content, making them flexible for use in downstream tasks.

LLMs have garnered significant attention in the medical domain [15]. Jiang et al. [37] developed the clinical LLM NYUTron to assist physicians, achieving AUC scores between 78.7 and 94.9%. It has been deployed in a prospective trial, indicating its potential for real-world application. In psychology, LLMs have also been applied to analyze suicidality, for instance by identifying linguistic markers [38], detecting risk cues [39], or extracting evidence from text [21,40]. However, a significant limitation of these pioneering works is their predominant focus on English-language data from platforms like Reddit, leaving the nuances of mental health expression in other cultural and linguistic contexts, such as Chinese social media, largely unexplored.

Other studies have explored LLMs as simulators for psychiatrist–patient interactions [41] or as support systems for non-professional counselors [42]. While insightful, these applications often rely on general-purpose models like ChatGPT, whose performance on specific, high-stakes psychological tasks requires deeper validation. For instance, a recent evaluation by Xu et al. [16] benchmarked several LLMs on English mental health datasets. Crucially, their study was confined to English and did not include complex multi-label classification, which is essential for phenomena like cognitive distortions, where multiple issues co-occur. Yang et al. [43] similarly found that while ChatGPT surpasses traditional methods, it still lags behind advanced, task-specific approaches, highlighting the need for more rigorous, domain-specific evaluations.

In summary, the existing literature, while promising, reveals two critical gaps that this paper aims to fill: (1) a scarcity of research focused on non-English languages like Chinese, and (2) a lack of systematic benchmarks on complex, clinically grounded tasks such as multi-label cognitive distortion detection. Our work directly confronts these limitations by introducing two novel Chinese datasets and providing a comprehensive benchmark analysis.

## 3. Methods

**Figure 1:**
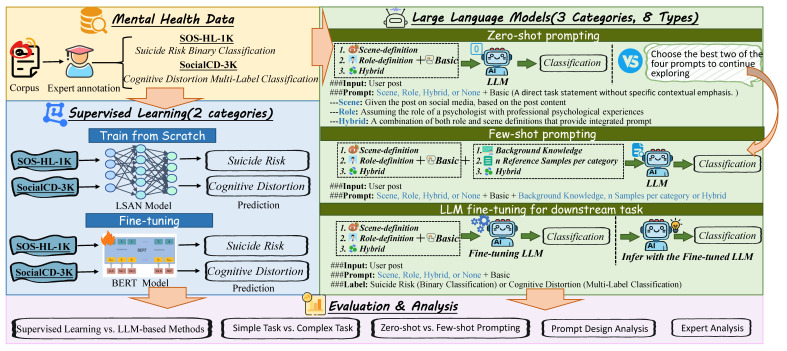
Workflow of our framework. In the figure, the thick, light-orange arrows represent the main data flow, while the dark green arrows indicate decision steps or inference loops in our experimental design.

To comprehensively evaluate the capabilities of LLMs in psychologically relevant classification tasks, we construct a multi-stage comparison framework that encompasses traditional supervised learning, zero-shot and few-shot prompt-based inference, and fine-tuning of LLMs. Specifically, we focus on two tasks derived from Chinese social media: binary suicide risk classification and multi-label cognitive distortion detection. Our framework contrasts two supervised baselines with eight LLMs, including both general-purpose and Chinese-adaptive models, under multiple prompting strategies. We design a series of zero-shot and few-shot prompts tailored to psychological scenarios, and further explore fine-tuning techniques to enhance LLM performance. The overall goal is to understand the strengths and limitations of different approaches in this domain and to provide empirical guidance for practical deployment. The structure of our framework is illustrated in Figure 1.

### 3.1. Baseline Supervised Learning Models

- LSAN [44]: The LSAN model uses label semantics to identify relationships between labels and documents, creating a label-specific document representation. It employs a self-attention mechanism to focus on this representation and uses an adaptive fusion strategy for multi-label text classification, proving effective in predicting low-frequency labels.
- BERT [45]: BERT uses a bidirectional approach, facilitated by the Transformer architecture [46], to understand the full context of words. It is pre-trained with a masked language model objective, predicting masked words. BERT excels in various NLP tasks, such as question answering and sentiment analysis, especially when fine-tuned on specific data.

### 3.2. Large Language Models

For the LLM comparison experiments, we evaluated eight popular models, including two general LLMs (GPT-3.5 and GPT-4 [47]) and six Chinese-adaptive LLMs (ChatGLM2-6B, GLM-130B [48], GLM-4 [49], Chinese-Alpaca-2-7B [50], Chinese-LLaMA-2-7B [50], and LLaMA2-Chinese-7b-Chat [51]).

#### 3.2.1. General LLMs: GPTs

- GPT-3.5: GPT-3.5 is an advanced iteration of the GPT-3 [52] language model, offering improvements in conversational capabilities. It is designed to provide more coherent and context-aware responses in conversational applications, reflecting ongoing developments in language modeling techniques.
- GPT-4 [47]: GPT-4 is a groundbreaking multimodal model that processes both images and text to generate text outputs. It performs at a human level on various benchmarks, including scoring in the top 10% on a simulated bar exam. Built on the Transformer architecture [46], GPT-4 is trained to predict tokens and undergoes post-training alignment for improved accuracy. Despite its capabilities, GPT-4 has limitations like occasional content hallucinations and a constrained context window.

#### 3.2.2. Chinese LLMs

- ChatGLM2-6B: ChatGLM2-6B is an open-source bilingual model with 6.2 billion parameters, optimized for Chinese question-answering and dialogue. Trained on about 1 TB of Chinese and English text, it can be fine-tuned through various techniques like supervised learning and human feedback, allowing for diverse language processing applications.
- GLM-130B [48]: GLM-130B is a bilingual model with 130 billion parameters, optimized for English and Chinese. It aims to provide an open-source alternative comparable to GPT-3, outperforming GPT-3 175B on multiple English benchmarks and surpassing ERNIE TITAN 3.0 260B [53] on Chinese benchmarks.
- GLM-4 [49]: GLM-4 uses a bidirectional Transformer [46] structure with the Masked and Prefix Language Model (MPCT) pre-training strategy, combining MLM and PLM strengths. It employs Rotary Position Embedding (RoPE) for long text sequences and adaptive masking for varied tasks, and supports multi-task learning.
- Chinese-LLaMA-2-7B [50]: Chinese-LLaMa-2-7B, based on LLaMa-2 [54], uses a new vocabulary of 55,296 entries to enhance Chinese text coverage. It employs Low-Rank Adaptation (LoRA) [55] for fine-tuning, supporting multi-task learning, and demonstrating superior performance in Chinese tasks.
- Chinese-Alpaca-2-7B [50]: Chinese-Alpaca-2-7B focuses on conversational and instruction-following capabilities. Using LoRA [55] for efficient training, it excels in multi-turn dialogues and complex command parsing after supervised fine-tuning.
- LLaMA2-Chinese-7b-Chat [51]: Built on LLaMA-2 [54], LLaMA2-Chinese-7b-Chat is optimized for Chinese through continuous pre-training on a large-scale dataset. It leverages LoRA [55] for training, enhancing conversational capabilities with Chinese-specific commands and data.

### 3.3. LLM Prompt Strategies

LLMs use prompts as guides and are valued for their flexibility in performing downstream tasks. However, with LLMs it is crucial to design the prompts precisely, as this directly affects its understanding of the given task and the output of the results [56]. To this end, we design the following prompt strategies, including zero-shot and few-shot approaches. Zero-shot prompts denote direct interaction with the LLM without providing any task examples, while few-shot prompts involve interaction with the LLM by giving some examples within tasks, similar to the training data used in the supervised learning.

#### 3.3.1. Zero-Shot Prompting

We begin our experiment with a prompt design tailored to tasks within a zero-shot paradigm, and it includes several strategies: direct task requests (represented as basic), role definition, scene definition, and a hybrid strategy. For illustrative purposes, the cognitive distortion classification task is used as an example. Note that, since our experimental data are in Chinese, we also use Chinese prompts. We provide translations of the English prompts in the following explanation. To avoid repetitions in prompts, we select the key part of each strategy and represent it as Strategy [Prompt]. When the prompt is a combination of several strategies from 1 to *n*, it is referred to as ${\mathrm{Strategy}}_{1}+{\mathrm{Strategy}}_{2}+\ldots +{\mathrm{Strategy}}_{n}$.

1. Basic: A direct task statement without specific contextual emphasis. Prompt English translation: “Basic [Please perform a multi-label classification task to determine whether it reflects any of the specified 12 cognitive distortions: (list of cognitive distortions categories)].”
2. Role-definition prompting: This strategy delineates the role of the LLM (in this case, a psychologist) and emphasizes the need for psychological expertise. Prompt English translation: “Role [Assuming the role of a psychologist with professional psychological experiences] +Basic”
3. Scene-definition prompting: Introduces the context of a social media environment, highlighting user identifiers to eliminate ambiguity. Prompt English translation: “Scene [Given the user ID *u* and the associated posts on social media, based on the post content], +Basic”
4. Hybrid prompting: A combination of both role and scene definitions that provides an integrated prompt, and it can be represented as Scene+Role+Basic.

#### 3.3.2. Few-Shot Prompting

Few-shot prompting is construed as a method to integrate prior knowledge or a batch of *n* training samples into LLMs, thereby enabling them to learn this information and adeptly execute the task. We experiment with the following setup.

1. Background knowledge: This strategy is provided with psychological definitions, supplemented by representative cases, followed by one of the four prompting strategies devised from zero-shot prompting. Prompts that integrate background knowledge and employ the hybrid strategy from zero-shot prompting are detailed as follows: “Background [There are the definitions of 12 cognitive distortions (list of cognitive distortions definitions), and there are the representative cases (list of cognitive distortions cases). Consider these cognitive distortions definitions and cases], +Scene+Role+Basic.”
2. Prompting with *n* reference samples per category: In this strategy, reference data ${\mathrm{ref}}_{n}$ are randomly selected from the training set for each category to construct prompts for the LLM, followed by one of the four prompting strategies designed from zero-shot prompting. Prompts that incorporate the reference data and employ the hybrid strategy from zero-shot prompting are detailed as follows: “Reference [There are some examples of the target task with the ground truth label (list of reference samples). Consider these cognitive distortions examples], +Scene+Role+Basic.”
3. Background knowledge and prompting with *n* reference samples per category: This approach investigates whether enhancing explanations in few-shot prompting can improve the LLM in terms of its understanding of psychological tasks. It incorporates psychological definitions, and provides *n* reference samples per category for LLM prompt conduction. The following example integrates background knowledge and reference instances, and employ the hybrid strategy from zero-shot prompting. It can be represented as Background+Reference+Scene+Role+Basic.

### 3.4. LLM Fine-Tuning for Downstream Task

Fine-tuning LLM is the process of adapting a pre-trained language model to a specific task or domain by continuing its training on a smaller, task-specific dataset [57]. Fine-tuning typically involves updating the model’s weights using supervised learning with labeled data, enhancing its performance in the targeted downstream task. Following the fine-tuning, our evaluation paradigm retained the role, scene, and hybrid definitions from the zero-shot prompting for consistency and comparative assessment as introduced in Section 3.3.1.

## 4. Experiments and Results

### 4.1. Datasets and Evaluation Metrics

We proposed two psychology-related classification datasets for high/low suicide risk classification and cognitive distortion multi-label classification, named “SOS-HL-1K” and “SocialCD-3K”. The suicide risk task primarily differentiates between high and low suicide risks, while the cognitive distortion task focuses on the classifications defined by Burns [20]. We sourced our data by crawling comments from the “Zoufan” blog within the Weibo social platform, a known space for users to express psychological distress, covering the period from 1 January 2017, to 31 December 2019. To ensure data relevance and quality, we first identified comments using keywords such as “depression”, “suicide”, and “despair”. Following this, all candidate posts underwent a manual review and cleaning process. We specifically excluded (1) positive and encouraging expressions, and (2) commercial advertisements, spam, and purely symbolic emoji comments. This multi-step preprocessing ensured our final datasets are composed of high-quality, genuine expressions of personal struggle suitable for psychological analysis.

To ensure the quality and reliability of our annotations, we employed three annotators with backgrounds in clinical psychology or counseling. Prior to annotation, all annotators underwent unified training using a detailed guide based on Burns’ cognitive distortion framework to ensure a shared understanding of each category’s definition. Each post in our dataset was independently annotated. To measure inter-rater reliability (IRR), we randomly sampled 20% of the data from both datasets for double-blind annotation. For the binary suicide risk classification task (SOS-HL-1K), we calculated Fleiss’ κ, achieving a score of 0.82, which indicates strong agreement. For the more complex multi-label cognitive distortion task (SocialCD-3K), we computed Krippendorff’s α, resulting in a value of 0.78, reflecting substantial agreement. Disagreements during the annotation process were resolved using a discussion-based strategy. If the initial annotators could not reach a consensus, a senior annotator, a licensed psychologist with over five years of clinical experience, served as the final arbitrator. After establishing this robust annotation standard, the remaining 80% of the data was annotated by the team. To ensure transparency and support future research, the detailed annotation guide, which includes definitions and representative examples for each category, is publicly available at https://github.com/HongzhiQ/SupervisedVsLLM-EfficacyEval (accessed on 28 May 2024).

The suicide detection dataset is a binary classification dataset consisting of 1249 samples. Among these, there are 648 posts with low suicide risk and 601 posts with high suicide risk. The cognitive distortion dataset contains a total of 3407 posts. This is a multi-label classification dataset, with each sample corresponding to one or more cognitive distortion categories. The classification labels used for this data follow the definitions provided by Burns [20]. For both sets of data, the training set and test set are divided according to the ratio of 4:1. The data distribution and specific categories of these two datasets are listed in Table 1. We utilize three primary evaluation metrics to measure the performance of different algorithms for our two tasks: precision, recall, and F1-score. Given the distinct nature of the two classification tasks, for the binary suicide risk classification task, we report micro-averaged and macro-averaged metrics as well as the specific precision, recall, and F1-score for the high-risk class. We emphasize the F1-score of the high-risk class in our main findings, as this positive class is the primary target for clinical intervention and early detection. For the multi-label cognitive distortion task, we report both micro-averaged and macro-averaged F1-scores to provide a comprehensive view of model performance. In line with best practices for imbalanced datasets, we primarily highlight the micro-averaged F1-score in our analysis. This method aggregates the contributions of all classes to compute an overall score, which is particularly useful in scenarios with a skewed distribution of class instances. The macro-averaged F1-score is presented as a complementary metric, offering insights into the model’s unweighted average performance across all categories, treating each class equally regardless of its frequency.

### 4.2. Experiment Design

Our experiments focus on validating the two proposed tasks, particularly testing different strategies for using LLMs. This includes four zero-shot and three few-shot prompt construction strategies, as well as the performance of fine-tuned LLMs. In total, we compare two types of supervised learning methods and eight LLMs. Using cognitive distortions as an example to show our points, our evaluations spanned several dimensions:

- Prompt design: Initially, we assessed four prompting strategies within the zero-shot learning conduction. Subsequently, based on their performance, the top two strategies were selected for further evaluation in the few-shot learning setting across various LLMs.
- LLM ability: In our experiments, we found that the performance of the ChatGLM2-6B and GLM-130B models performed poorly. Therefore, we decided to use the latest version of the GLM series, the GLM-4 model, to evaluate performance on the newly expanded dataset. For GPT-3.5, its token limitation prevented us from entering five samples for each category during few-shot prompting. Consequently, we reserved the ${\mathrm{ref}}_{5}$ approach exclusively for GPT-4.
- LLM fine-tuning: OpenAI recently introduced a fine-tuning feature for GPT-3.5, and official reports suggest that, under certain conditions, fine-tuned GPT-3.5 may outperform GPT-4 [58]. Therefore, we experimented with fine-tuning GPT-3.5. Since the current version of GPT-4 lacks fine-tuning capabilities, we were unable to assess its potential. Additionally, we explored the fine-tuning performance of three other open-source Chinese large models in the cognitive distortion recognition task.

Note that, since the experimental performance of the three Chinese LLMs (Chinese-Alpaca-2-7B, Chinese-LLaMA-2-7B, and LLaMA2-Chinese-7b-Chat) on cognitive distortion was similar to the performance of GPT-3.5 after fine-tuning, we did not conduct experiments with these three LLMs in the suicide risk classification task. Also, ChatGLM2-6B’s performance in the cognitive distortion classification task was too poor to even output in the correct format, so we did not include its experimental results. By the time we experimented with the cognitive distortion task, GLM-130B had been replaced by a newer version, GLM-4, so we evaluated GLM-4 in this task instead.

### 4.3. Implementation Details

Prior to model input, all text underwent a standardized data preprocessing pipeline. First, to ensure user privacy and data cleanliness, personally identifiable information such as usernames was masked, and all URLs were removed. Subsequently, all emojis were removed from the text. To preserve the full contextual integrity required by modern language models, we deliberately retained stopwords. Finally, the preprocessed text was fed into each respective model, where its native tokenizer automatically performed the necessary steps, including tokenization, conversion to numerical IDs, and the addition of special tokens (e.g., [CLS] and [SEP]) to convert the text into the required input format.

- LSAN: We used Word2vec [59] to train 300-dimensional embeddings for both the document and randomly initialized label texts. The function of the attention mechanism is to compute word contributions to labels and create label-specific document representations. Dot products between these document and label vectors refined these relationships further. These two types of document representations were then fused using weighted combinations. For predictions, we employed a fully connected layer, followed by ReLU and a sigmoid function. We used cross-entropy as the loss function.
- BERT: We employed a Chinese pre-trained BERT model to extract 768-dimensional vectors from the sentences. To avoid overfitting, a dropout function [60] was applied to these sentence vectors. Subsequently, a fully connected layer was introduced for classification. The sigmoid function served as the activation function for the output layer.
- LLM-zero shot: Both GPT-3.5 and GPT-4 [47] are closed-source and available through OpenAI’s API. We used Gpt-3.5-turbo because it is one of the most capable and cost-effective models in the GPT-3.5 family, and GPT-4 for its advanced capabilities. For GLM models, we deployed the open-source ChatGLM2-6B [48] on our server, and tested the larger GLM-130B [48] via its official website due to deployment challenges. We averaged the performance over five rounds of experiments for all models to minimize randomness. For GPT-3.5, GPT-4, ChatGLM2-6B, and GLM-4, we set the temperature to 0.1, 0.3, 0.5, 0.7, and 0.9, and averaged the results over five rounds of experiments. For GLM-130B, we could not adjust the temperature due to its limitations.
- LLM-few shot: We conducted the experiments using the top two performing prompt strategies from the zero-shot performance as determined by their F1-scores. Given the different input token constraints of each model, we selected varying amounts of reference data ${\mathrm{ref}}_{n}$ according to the requirements of the corresponding models.
- LLM-fine-tuning: For the closed-source model GPT-3.5, we used the API provided by OpenAI to fine-tune the GPT-3.5 Turbo model for suicide risk and cognitive distortions tasks. In this experiment, the training epoch was set to 3. For other hyperparameters, we did not explicitly specify them; instead, OpenAI selected the default values based on the dataset size. We also explored the performance of three open-source Chinese LLMs (Chinese-LLaMA-2-7B, Chinese-Alpaca-2-7B, and LLaMA2-Chinese-7b-Chat) on this fine-tuning task. These models were fine-tuned locally on an NVIDIA A100 GPU. The training process was configured with a batch size of 8, a learning rate of $6\times 10^{-5}$ using a cosine scheduler, and a weight decay of 0.01, and was run for 5 epochs, with model checkpoints saved every 300 steps. We utilized Low-Rank Adaptation (LoRA) for parameter-efficient fine-tuning. The LoRA-specific configuration was set to a rank of 8, an alpha of 32, and a lora_dropout rate of 0.05. The LoRA modules were applied to the attention layers’ query and value projection matrices (q_proj and v_proj). To ensure full reproducibility, the complete configuration file for these experiments is available in our GitHub repository.

To ensure the robustness and reproducibility of our findings, all experiments were conducted multiple times. For the supervised learning models, we performed five independent runs using different random seeds (42, 52, 62, 72, and 82) for data shuffling and model initialization, and reported the average and standard deviation of the results. For all LLM-based evaluations (zero-shot, few-shot, and fine-tuning), we conducted experiments under five different temperature settings (0.1, 0.3, 0.5, 0.7, and 0.9). The final reported LLM scores were reported as the mean and standard deviation across these five runs. This approach allows us to assess the stability of the supervised models against random initialization and the sensitivity of the LLMs to the temperature hyperparameter. To assess the statistical significance of performance differences between experimental conditions, we conducted paired-samples *t*-tests with a significance level of α=0.05.

To ensure transparency and reproducibility, we detail the computational environment used for our experiments. All local experiments were conducted on a workstation equipped with a single NVIDIA A100 GPU (40 GB VRAM) and 256 GB of RAM. For supervised learning models (e.g., BERT and LSAN), the average fine-tuning time was approximately 1 h. Fine-tuning LLMs with LoRA (e.g., Chinese-Alpaca-2-7B and LLaMA2-Chinese-7b-Chat) required between 3 and 5 h depending on the model size. For GPT-3.5, fine-tuning was performed via OpenAI’s API service. Due to API rate limits and network latency, each run took approximately 4 to 5 h to complete. Although not GPU-bound locally, GPT-3.5 fine-tuning was the most time consuming among all models. All zero-shot and few-shot evaluations were completed within seconds per instance and conducted using the same A100 GPU, except for GPT-3.5 and GPT-4, which were accessed via the OpenAI API.

## 5. Results

### 5.1. Effect of Prompting Strategies on Task Performance

In our study, we primarily aimed to answer two questions: how can LLMs be most simply used to accomplish a task, and what is the performance potential of LLMs on both tasks? The order of using LLMs ranges from simple to complex, starting with zero-shot and few-shot prompt engineering to the fine-tuning. This analysis is detailed in the following sections, and the results of suicide classification and cognitive distortions classification can be seen in Table 2 and Table 3, respectively.

In the zero-shot prompting strategy, model performance exhibited a clear dependence on task complexity. For the relatively simple suicide risk classification task, all LLMs, except for the smaller ChatGLM2-6B, achieved F1-scores close to 70% on the high-risk class. As shown in Table 2, the performance gap among the top-performing models was small. GPT-4, using the scene-definition strategy, achieved an average F1-score of 73.39% for high-risk detection, slightly outperforming the second-best GLM-130B (72.20%, hybrid strategy). Compared to other models, GPT-4 significantly outperformed GPT-3.5 (68.59%, t=7.18, p<0.001) and ChatGLM2-6B (53.74%, t=4.59, p<0.01). Although GPT-4 achieved the highest mean F1-score among all models, its difference from GLM-130B was not statistically significant, indicating that GLM-130B is a competitive alternative in the zero-shot setting. Overall, the best-performing GPT-4 and GLM-130B models showed micro-F1 and macro-F1-scores that were close to each other but generally lower than their high-risk class F1 (by approximately 1–8 percentage points), suggesting that while overall and macro-averaged performance was stable, high-risk sample detection remained the most discriminative indicator.

The importance of prompt engineering was also validated in this experiment. Different models benefited from different prompt construction strategies; for example, the hybrid strategy worked best for GLM-130B, whereas the role-definition strategy was optimal for GPT-3.5. Notably, for GPT-4, its best prompt strategy (scene-definition) outperformed its weakest strategy (basic) by approximately 1.67 percentage points in average F1, but the difference did not reach statistical significance (p=0.094). This suggests that prompt design can sometimes improve performance but may yield limited and unstable gains. However, zero-shot prompting proved inadequate for the more complex cognitive distortion multi-label classification task. The best-performing zero-shot model, GLM-4 (role-definition strategy), achieved a micro-F1 of only 28.59%, with an even lower macro-F1 of 22.11%. Compared to the supervised BERT model (micro-F1: 76.10%, macro-F1: 70.05%), this represents substantial performance gaps of 47.51 and 47.94 percentage points, respectively, both highly significant (micro-F1: t=57.40, p<0.001; macro-F1: t=44.68, p<0.001). Furthermore, the significantly lower macro-F1 compared to micro-F1 (e.g., GPT-4: t=5.10, p<0.01) can be attributed to the downward pull from long-tail categories in the evaluation phase. Some categories had very few test samples, making their F1-scores highly sensitive to single prediction successes or failures, often dropping close to zero with minor misclassifications. Consequently, while zero-shot prompting may suffice for well-defined binary classification tasks, it falls far short for complex multi-label reasoning tasks like cognitive distortion detection.

The few-shot prompting strategy involves incorporating a small number of labeled examples into the prompt as references to guide the model in completing the task. In the suicide risk classification task, this approach significantly improved upon the zero-shot baseline. For example, GPT-4’s high-risk class mean F1 increased from 73.39% to 75.81% (t=2.63, p<0.05), with both micro-F1 and macro-F1 increasing by 8.88 and 12.68 percentage points, respectively, each reaching statistical significance (micro-F1: t=6.37, p<0.01; macro-F1: t=5.72, p<0.01). For ChatGLM2-6B, this strategy yielded an 11.04 percentage point improvement in high-risk F1, with the best result under the background + ref_12_ + hybrid setting. However, increasing the example count to ${\mathrm{ref}}_{30}$ did not further improve performance and instead caused a decline (F1 from 64.78% to 51.88%), though this drop was not statistically significant (p>0.05), suggesting that overly long contexts may introduce distracting information and hinder reasoning. In the cognitive distortion multi-label classification task, overall performance under few-shot settings remained low (best F1 only 43.78%) but still improved significantly compared to zero-shot. For example, GLM-4’s micro-F1 increased by 15.19 percentage points (p<0.001), and GPT-4’s micro-F1 improved by 13.78% (t=26.05, p<0.001). In summary, few-shot prompting can deliver statistically significant gains across tasks, but the improvement magnitude strongly depends on the initial performance baseline: for tasks already performing well (e.g., suicide risk classification), gains are modest but consistent; for low-baseline, complex reasoning tasks (e.g., cognitive distortion detection), improvements are much larger.

Fine-tuned LLMs achieved better performance in both tasks. In the suicide classification task, the fine-tuned GPT-3.5 model significantly outperformed the zero-shot model, improving high-risk F1 by 9.86 percentage points (t=63.02, p<0.001). In the cognitive distortion task, the performance gain was even more pronounced: compared with the best zero-shot prompt strategy, the fine-tuned GPT-3.5’s micro-average F1 improved dramatically (mean: 72.21% vs. 12.06%), with the difference being highly significant (t=76.01, p<0.001). However, as the performance gaps among fine-tuned LLMs in the cognitive distortion task were small, no further fine-tuned LLM comparisons were conducted for the suicide risk classification task. Despite the substantial improvements from fine-tuning, supervised learning maintained a performance advantage in both tasks. In the suicide classification task, BERT achieved the highest high-risk F1-score of 82.76%, surpassing the best fine-tuned GPT-3.5 model (mean: 78.45%), with the difference confirmed as statistically significant (t=53.52, p<0.001). Similarly, in the cognitive distortion detection task, BERT outperformed all LLM-based methods, achieving a micro-F1 of 76.10% compared to 72.96% for the best LLM (Chinese-Alpaca-2-7B), a difference that was also statistically significant (t=26.71, p<0.001). More importantly, we observed that all models achieved much higher micro-F1-scores than macro-F1-scores in this task (e.g., BERT: 76.10% vs. 70.05%; Chinese-Alpaca-2-7B: 72.96% vs. 66.89%). This significant gap indicates that due to the imbalanced distribution of cognitive distortion categories in the dataset, models performed well on frequent categories but poorly on rare ones. Micro-F1, being dominated by large classes, was thus inflated, whereas macro-F1, giving equal weight to all classes, exposed the models’ shortcomings in handling long-tail categories. Furthermore, while task-specific fine-tuning can enhance LLM performance, it still cannot surpass supervised learning. Fine-tuning also increases LLM usage complexity and cost, reducing flexibility and versatility. LLMs typically have far more parameters than supervised models, making them more expensive to train and deploy.

Overall, LLMs are effective for performing relatively simple tasks using only zero-shot prompt, but they need to be properly designed with prompt such as scene settings. For more difficult tasks, LLMs cannot perform adequately through prompt-only solution. The few-shot strategy, which introduces training data into the prompt, is effective but does not result in a significant performance improvement. Fine-tuning LLMs for specific tasks can achieve substantial improvements, but their performance still falls short of that achieved by supervised learning. Supervised learning remains the best choice for low-cost and high-performance in domain-specific tasks.

### 5.2. Temperature Sensitivity Analysis

**Figure 2:**
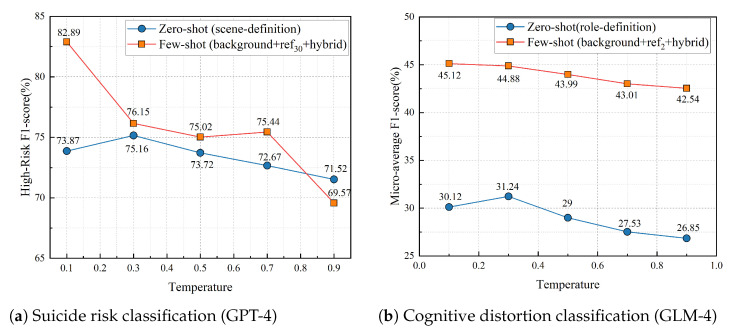
Effect of decoding temperature on model performance for the best LLM settings in each task.

To examine the effect of decoding temperature on model performance, we analyzed the results across different temperature values (T∈0.1,0.3,0.5,0.7,0.9) for both the zero-shot and few-shot settings of the best-performing LLM in each task as illustrated in Figure 2. For the suicide risk classification task, GPT-4 under the zero-shot scene-definition setting achieved its highest high-risk F1-score at T=0.3 (75.16%), with stable performance for T≤0.5 and a gradual decline thereafter (T=0.9, 71.52%). In the few-shot background+ref_3_0+hybrid setting, performance peaked at T=0.1 (82.89%), dropped markedly at T=0.5 (75.02%), and remained moderate for higher temperatures. Notably, at T=0.9, the score decreased substantially to 69.57%, indicating that few-shot prompting becomes less stable when the temperature is set too high, likely due to increased randomness in token sampling.

For the cognitive distortion multi-label classification task, GLM-4 in the zero-shot role-definition setting showed its best micro-average F1 at T=0.3 (31.24%), with a consistent downward trend as temperature increased. In the few-shot background + ref_2_ + hybrid setting, performance was highest at T=0.1 (45.12%) and decreased steadily to 42.54% at T=0.9. These results indicate that lower temperatures (0.1–0.3) generally yield better and more stable classification outcomes for both tasks, while higher temperatures introduce greater output variability and reduced accuracy, consistent with the precision requirements of psychological risk detection.

## 6. Expert Evaluation and Feedback

**Figure 3:**
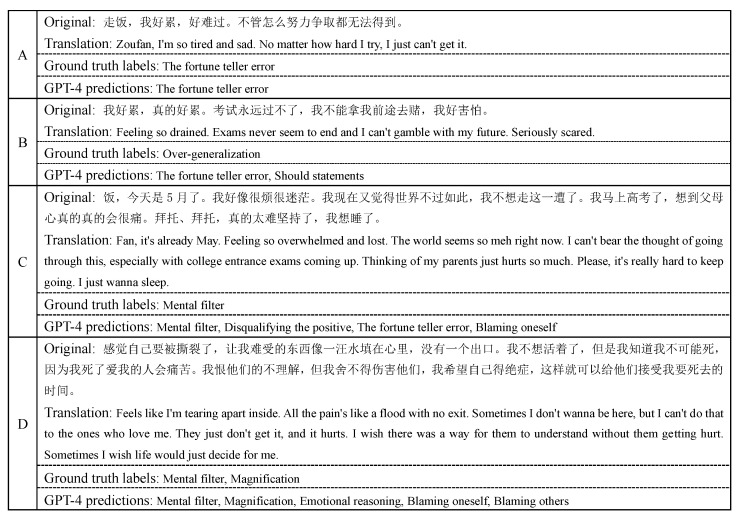
Examples of expert annotated labels and GPT-4 predictions in cognitive distortion classification task.

As mentioned above, using LLMs with prompts alone, without fine-tuning, is the best case scenario for their role. However, LLMs do not perform well under zero-shot and few-shot prompts. For this reason, we selected the output of GPT-4, which performs relatively well under the “${\mathrm{ref}}_{2}$ + basic” strategy, for further analysis. The examples can be seen in Figure 3.

Overall, the model’s judgments tend to be overly predictive and overly sensitive. When dealing with simple texts and scenarios, such as in Example A, the model can often make accurate predictions. However, simplicity does not necessarily imply clarity; in fact, seemingly simple statements may contain ambiguous or implicit meanings that are more difficult to evaluate. For instance, in Example B, the model identifies negative expectations regarding exam outcomes and associated emotions, classifying the text under “the fortune teller error” and “should statements”. However, it fails to capture the core cognitive pattern: the user’s generalized conclusion based on past failures. A more appropriate categorization would be “overgeneralization”, as the writer extrapolates that “the exams will never be passed” based on a limited number of unsuccessful attempts.

These examples demonstrate that while the model can provide generally plausible classifications, it often misses the central semantic feature of the text, leading to mislabeling or incorrect predictions. When processing longer and more complex texts, as in Examples C and D, the model often succeeds in identifying one or more correct categories but exhibits a consistent tendency toward over-prediction. In Example C, the model correctly labels the sentence with “mental filter” but also incorrectly assigns three additional categories: “disqualifying the positive”, “the fortune teller error”, and “blaming oneself”. These false positives are not substantiated by the content—there is no explicit denial of positive experiences, no exaggerated future prediction, nor any self-blaming element. Similarly, in Example D, the model correctly identifies “mental filter” and “magnification”, reflecting the user’s focus on negative details and exaggeration of the problem’s severity. However, it further predicts three unsupported distortions—“emotional reasoning”, “blaming oneself”, and “blaming others”—none of which are explicitly expressed in the original text.

This over-prediction behavior may be influenced by the prompting strategy. The hybrid prompt, which frames the model as a psychologist, may lead to hyper-vigilant responses. In an effort to fulfill this role comprehensively, the model becomes overly sensitive to subtle cues, resulting in the identification of both well-supported distortions and additional categories based on weak or inferred signals. This is particularly evident in Example D, where the model appears to over-interpret the user’s emotional intensity, resulting in multiple false positives.

Overall, these examples highlight that while the model often aligns with the general direction of the user’s intent, it tends to over-interpret nuanced language, thereby reducing prediction precision. Future work should focus on enhancing the model’s ability to prioritize central semantic features over peripheral cues, which may help reduce over-prediction and improve overall classification accuracy.

## 7. Conclusions

In this paper, we present two psychologically relevant datasets based on social media data: SocialCD-3K for cognitive distortion multi-label classification, and SOS-HL-1K for high/low suicide risk classification. We validate the performance of two supervised learning models and eight LLMs, including both Chinese and English models, on these tasks. Our experiments evaluate the performance of LLMs under different prompt designs. The results show that prompt-only LLMs can perform reasonably well on the suicide risk classification task but fall short in more complex scenarios such as cognitive distortion recognition. While fine-tuning improves performance, it still underperforms compared to supervised learning methods. Additionally, fine-tuning may reduce the flexibility and ease of use of LLMs in deployment. Nevertheless, our study provides new research resources to the community and offers detailed experimental insights for applying LLMs in the mental health domain. The SocialCD-3K dataset could also support early-warning systems for suicide prevention hotlines by offering a rich source of annotated linguistic patterns associated with different levels of psychological distress. As future work, we will expand the datasets and conduct cross-platform and out-of-time evaluations, while exploring confidence calibration, selective prediction, and prompt-robustness strategies to reduce over-prediction and improve real-world generalization.

## Figures and Tables

**Table 1:** Data distribution of the proposed datasets: SOS-HL-1K and SocialCD-3K.

Dataset	Categories	$N_{c}$
SOS-HL-1K	High-risk	601
	Low-risk	648
	$N_{train}$ = 999, $N_{test}$ = 250, *L* = 1, $\bar{L}$ = 1, $\bar{C}$ = 47.79	
SocialCD-3K	All-or-nothing thinking	77
	Over-generalization	141
	Mental filter	378
	Disqualifying the positive	27
	Mind reading	121
	The fortune teller error	652
	Magnification	321
	Emotional reasoning	16
	Should statements	84
	Labeling and mislabeling	1961
	Blaming oneself	188
	Blaming others	27
	$N_{train}$ = 2725, $N_{test}$ = 682, *L* = 12, $\bar{L}$ = 1.71, $\bar{C}$ = 42.56	

Note. $N_{c}$: Number of posts in each category. $N_{train}$/$N_{test}$: Size of training/test set. *L*: Total number of label types. $\bar{L}$: Average number of labels per post. $\bar{C}$: Average number of words per post.

**Table 2:** Results for the suicide binary classification task. All these methods were evaluated on the same test set containing 250 posts. The terms “zero-shot” and “few-shot” refer to different prompt construction without training. “Train from scratch” and “fine-tuning” indicate that the corresponding method was trained or fine-tuned using the training set. Reported metrics include micro-averaged, macro-averaged, and high-risk class precision, recall, and F1-score, presented as mean (±standard deviation) over five runs.

Model Category	Model	Type	Sub-Type	Train Data	Micro-Average	Macro-Average			High-Risk		
					F1-Score (%)	Precision (%)	Recall (%)	F1-Score (%)	Precision (%)	Recall (%)	F1-Score (%)
Supervised learning	LSAN	Train from scratch	-	999	83.20 (±1.15)	82.75 (±1.05)	83.75 (±0.90)	82.81 (±0.98)	74.59 (±1.21)	87.50 (±0.95)	80.53 (±0.87)
	BERT	Fine-tuning	-	999	87.20 (±0.61)	87.41 (±0.65)	85.54 (±0.78)	86.23 (±0.68)	88.42 (±0.75)	77.78 (±1.02)	82.76 (±0.56)
LLM	ChatGLM2-6B	Zero-shot	basic	0	60.80 (±4.91)	63.41 (±5.32)	60.61 (±4.93)	58.26 (±5.99)	69.07 (±7.02)	37.10 (±8.55)	48.07 (±8.99)
			role	0	58.56 (±4.20)	60.94 (±5.81)	58.38 (±4.18)	56.19 (±3.97)	65.77 (±9.00)	35.81 (±4.25)	46.15 (±4.48)
			scene	0	60.56 (±3.22)	61.51 (±3.18)	60.44 (±3.24)	59.49 (±3.60)	64.52 (±3.57)	45.16 (±6.57)	53.01 (±5.33)
			hybrid	0	61.20 (±6.74)	62.39 (±7.34)	61.08 (±6.75)	60.04 (±7.12)	65.68 (±9.61)	46.13 (±10.90)	53.74 (±9.47)
		Few-shot	background + scene	0	57.44 (±6.28)	57.98 (±6.48)	57.36 (±6.34)	56.47 (±6.59)	58.56 (±6.23)	47.26 (±16.11)	51.45 (±10.71)
			background + hybrid	0	62.80 (±16.62)	64.29 (±18.11)	62.86 (±16.65)	62.23 (±16.60)	60.37 (±13.17)	70.64 (±23.99)	64.41 (±17.14)
			${\mathrm{ref}}_{12}$ + scene	24	65.68 (±6.41)	65.88 (±6.33)	65.63 (±6.43)	65.48 (±6.54)	67.19 (±6.20)	59.52 (±9.43)	63.04 (±7.88)
			${\mathrm{ref}}_{12}$ + hybrid	24	61.04 (±8.50)	61.75 (±9.08)	60.95 (±8.50)	60.38 (±8.58)	64.29 (±11.40)	49.20 (±9.18)	55.56 (±9.56)
			background + ${\mathrm{ref}}_{12}$ + scene	24	50.00 (±9.17)	50.09 (±9.80)	50.05 (±9.16)	49.53 (±9.26)	49.74 (±7.41)	56.61 (±12.12)	52.70 (±8.70)
			background + ${\mathrm{ref}}_{12}$ + hybrid	24	60.64 (±9.29)	61.57 (±9.99)	60.74 (±9.26)	59.51 (±10.15)	58.91 (±7.92)	73.23 (±11.86)	64.78 (±7.59)
			${\mathrm{ref}}_{30}$ + scene	60	55.28 (±8.32)	55.83 (±11.10)	55.05 (±8.36)	50.78 (±10.80)	57.71 (±16.53)	26.77 (±14.14)	36.14 (±16.74)
			${\mathrm{ref}}_{30}$ + hybrid	60	52.16 (±5.99)	51.27 (±8.31)	51.94 (±6.04)	47.57 (±8.85)	50.60 (±12.52)	24.84 (±13.85)	32.60 (±15.48)
			background + ${\mathrm{ref}}_{30}$ + scene	60	60.64 (±12.21)	61.33 (±12.79)	60.58 (±12.20)	60.23 (±12.07)	62.97 (±14.85)	52.90 (±13.93)	57.02 (±12.67)
			background + ${\mathrm{ref}}_{30}$ + hybrid	60	58.56 (±5.52)	59.11 (±5.37)	58.47 (±5.59)	57.41 (±6.39)	60.14 (±4.83)	47.10 (±16.21)	51.88 (±11.30)
	GLM-130B	Zero-shot	basic	0	58.32 (±2.37)	69.53 (±3.65)	58.62 (±2.35)	51.69 (±3.88)	54.58 (±1.49)	95.81 (±2.57)	69.52 (±1.36)
			role	0	59.68 (±2.79)	69.34 (±3.52)	59.96 (±2.77)	54.16 (±4.13)	55.51 (±1.77)	94.84 (±2.18)	70.02 (±1.66)
			scene	0	58.96 (±1.43)	68.04 (±4.36)	59.24 (±1.44)	53.53 (±1.07)	55.05 (±0.75)	93.87 (±3.20)	69.40 (±1.46)
			hybrid	0	62.80 (±0.49)	74.79 (±2.02)	63.07 (±0.49)	57.97 (±0.85)	57.37 (±0.35)	97.42 (±1.75)	72.20 (±0.47)
		Few-shot	background + role	0	60.80 (±1.85)	66.90 (±3.41)	61.03 (±1.86)	57.25 (±1.72)	56.55 (±1.10)	90.32 (±3.28)	69.55 (±1.77)
			background + hybrid	0	61.52 (±2.29)	69.00 (±3.86)	61.77 (±2.29)	57.62 (±2.73)	56.91 (±1.47)	92.42 (±3.45)	70.43 (±1.82)
			${\mathrm{ref}}_{12}$ + role	24	55.36 (±1.28)	58.17 (±2.47)	55.58 (±1.30)	51.78 (±0.88)	53.18 (±0.74)	83.23 (±3.29)	64.89 (±1.53)
			${\mathrm{ref}}_{12}$ + hybrid	24	58.80 (±0.57)	63.91 (±1.71)	59.03 (±0.58)	55.02 (±0.73)	55.30 (±0.32)	88.39 (±3.05)	68.02 (±0.97)
			background + ${\mathrm{ref}}_{12}$ + role	24	61.60 (±1.10)	64.44 (±1.05)	61.77 (±1.09)	59.80 (±1.32)	57.84 (±0.87)	83.38 (±0.44)	68.30 (±0.67)
			background + ${\mathrm{ref}}_{12}$ + hybrid	24	66.24 (±1.08)	71.07 (±0.71)	66.38 (±1.06)	64.29 (±1.50)	60.88 (±1.07)	90.00 (±1.47)	72.61 (±0.48)
	GPT-3.5	Zero-shot	basic	0	53.76 (±4.28)	57.82 (±7.62)	54.03 (±4.28)	47.76 (±4.81)	52.00 (±2.25)	88.23 (±1.98)	65.42 (±1.81)
			role	0	56.32 (±0.95)	67.70 (±2.39)	56.64 (±0.95)	48.44 (±1.59)	53.31 (±2.05)	96.13 (±1.55)	68.59 (±1.67)
			scene	0	54.08 (±2.57)	58.84 (±4.69)	54.36 (±2.58)	47.89 (±2.93)	52.16 (±2.11)	89.03 (±2.01)	65.76 (±1.94)
			hybrid	0	54.80 (±2.12)	61.06 (±3.34)	55.10 (±2.10)	47.69 (±3.36)	52.55 (±1.98)	92.26 (±1.82)	66.95 (±1.78)
		Few-shot	background + role	0	58.24 (±1.69)	63.49 (±2.95)	58.48 (±1.69)	54.17 (±2.04)	54.90 (±1.07)	88.55 (±3.84)	67.76 (±1.63)
			background + hybrid	0	58.80 (±1.67)	64.06 (±2.06)	59.04 (±1.67)	54.85 (±2.11)	55.27 (±1.14)	89.03 (±1.22)	68.19 (±1.09)
			${\mathrm{ref}}_{12}$ + role	24	59.76 (±1.37)	62.98 (±1.69)	59.95 (±1.37)	57.50 (±1.52)	56.34 (±0.96)	83.39 (±1.51)	67.22 (±1.08)
			${\mathrm{ref}}_{12}$ + hybrid	24	60.96 (±1.37)	64.29 (±1.69)	61.15 (±1.37)	58.77 (±1.52)	57.19 (±0.96)	84.68 (±1.51)	68.27 (±1.08)
			background + ${\mathrm{ref}}_{12}$ + role	24	63.20 (±1.57)	65.53 (±2.62)	63.35 (±1.59)	61.98 (±1.31)	59.37 (±0.83)	81.61 (±4.50)	68.71 (±2.04)
			background + ${\mathrm{ref}}_{12}$ + hybrid	24	62.08 (±2.25)	64.86 (±3.31)	62.25 (±2.26)	60.46 (±2.14)	58.26 (±1.44)	82.90 (±4.13)	68.41 (±2.26)
		Fine-tuning	role	999	83.12 (±0.55)	82.25 (±0.49)	81.44 (±0.72)	81.80 (±0.58)	84.76 (±0.51)	71.77 (±0.98)	77.73 (±0.42)
			scene	999	83.36 (±0.51)	82.81 (±0.56)	82.02 (±0.69)	82.40 (±0.54)	84.11 (±0.63)	72.58 (±0.85)	77.92 (±0.49)
			hybrid	999	83.60 (±0.48)	83.83 (±0.55)	82.05 (±0.61)	82.70 (±0.45)	84.26 (±0.45)	73.39 (±0.81)	78.45 (±0.38)
	GPT-4	Zero-shot	basic	0	62.64 (±2.05)	72.38 (±3.40)	62.90 (±2.05)	58.33 (±2.57)	57.43 (±1.31)	95.48 (±2.10)	71.72 (±1.42)
			role	0	62.64 (±2.74)	74.26 (±3.90)	62.91 (±2.73)	57.77 (±3.50)	57.29 (±1.73)	97.26 (±2.03)	72.10 (±1.82)
			scene	0	64.88 (±2.11)	76.03 (±2.59)	65.14 (±2.09)	60.86 (±2.68)	58.81 (±1.46)	97.58 (±1.51)	73.39 (±1.37)
			hybrid	0	62.96 (±2.60)	74.67 (±4.57)	63.23 (±2.60)	58.22 (±3.02)	57.47 (±1.57)	97.42 (±2.38)	72.30 (±1.89)
		Few-shot	${\mathrm{ref}}_{12}$ + scene	24	66.88 (±1.21)	74.28 (±1.59)	67.10 (±1.21)	64.32 (±1.52)	60.70 (±0.93)	94.35 (±1.51)	73.87 (±0.83)
			${\mathrm{ref}}_{12}$ + hybrid	24	64.00 (±3.36)	66.96 (±4.09)	64.16 (±3.35)	62.52 (±3.60)	59.77 (±2.60)	84.19 (±4.51)	69.87 (±2.75)
			background + ${\mathrm{ref}}_{12}$ + scene	24	69.28 (±5.60)	70.58 (±5.11)	69.38 (±5.56)	68.74 (±6.05)	65.44 (±5.37)	81.77 (±2.77)	72.63 (±4.07)
			background + ${\mathrm{ref}}_{12}$ + hybrid	24	68.96 (±1.99)	69.85 (±1.78)	69.04 (±1.97)	68.65 (±2.14)	65.65 (±2.48)	78.87 (±2.70)	71.60 (±1.36)
			${\mathrm{ref}}_{30}$ + scene	60	67.04 (±1.56)	73.13 (±1.97)	67.24 (±1.55)	64.87 (±1.98)	61.11 (±1.28)	92.42 (±2.32)	73.56 (±1.09)
			background + ${\mathrm{ref}}_{30}$ + hybrid	60	73.76 (±5.63)	74.59 (±5.41)	73.83 (±5.61)	73.54 (±5.77)	70.16 (±5.76)	82.58 (±4.21)	75.81 (±4.74)

**Table 3:** Results for the cognitive distortion multi-label classification task. All these methods were evaluated on the same test set containing 682 posts. The micro and macro metrics are presented as mean (±standard deviation) over five runs.

Model Category	Model	Type	Sub-Type	Train Data	Micro-Average			Macro-Average		
					Precision (%)	Recall (%)	F1-Score (%)	Precision (%)	Recall (%)	F1-Score (%)
Supervised learning	LSAN	Train from scratch	-	2725	75.53 (±0.85)	71.48 (±0.91)	73.45 (±0.88)	70.10 (±1.15)	67.00 (±1.21)	67.28 (±1.18)
	BERT	Fine-tuning	-	2725	85.43 (±0.68)	68.62 (±0.85)	76.10 (±0.45)	75.00 (±0.98)	68.30 (±1.15)	70.05 (±0.85)
LLM	GLM-4	Zero-shot	basic	0	17.39 (±2.41)	46.95 (±2.95)	25.38 (±2.68)	16.52 (±3.11)	45.15 (±3.65)	21.65 (±3.28)
			role	0	20.56 (±2.25)	46.95 (±2.81)	28.59 (±2.51)	19.88 (±2.95)	45.82 (±3.51)	22.11 (±3.21)
			scene	0	17.19 (±2.55)	45.33 (±3.08)	24.93 (±2.85)	16.81 (±3.25)	44.92 (±3.78)	21.05 (±3.55)
			hybrid	0	19.54 (±2.31)	47.32 (±2.88)	27.66 (±2.59)	18.95 (±3.01)	43.93 (±3.58)	21.67 (±3.29)
		Few-shot	background + hybrid	0	30.63 (±2.01)	48.82 (±2.15)	37.64 (±2.08)	25.50 (±2.61)	41.21 (±2.75)	25.30 (±2.68)
			background + role	0	28.76 (±2.11)	47.20 (±2.28)	35.74 (±2.18)	27.01 (±2.71)	43.43 (±2.88)	25.65 (±2.78)
			${\mathrm{ref}}_{2}$ + hybrid	24	32.25 (±1.85)	43.21 (±2.05)	36.94 (±1.95)	23.96 (±2.45)	37.05 (±2.65)	23.05 (±2.55)
			${\mathrm{ref}}_{2}$ + role	24	34.82 (±1.72)	44.58 (±1.91)	39.10 (±1.81)	23.64 (±2.32)	30.17 (±2.51)	22.40 (±2.41)
			background + ${\mathrm{ref}}_{2}$ + hybrid	24	41.74 (±1.55)	47.82 (±1.48)	44.57 (±1.51)	27.04 (±2.05)	40.96 (±1.98)	25.59 (±2.01)
			background + ${\mathrm{ref}}_{2}$ + role	24	41.91 (±1.51)	45.83 (±1.62)	43.78 (±1.58)	28.75 (±2.01)	34.27 (±2.22)	27.03 (±2.08)
			${\mathrm{ref}}_{5}$ + hybrid	60	26.32 (±2.58)	34.74 (±2.71)	29.95 (±2.65)	23.11 (±3.28)	29.98 (±3.41)	18.80 (±3.35)
			${\mathrm{ref}}_{5}$ + role	60	29.67 (±2.45)	36.61 (±2.65)	32.78 (±2.52)	23.72 (±3.15)	32.71 (±3.35)	20.74 (±3.22)
	GPT-3.5	Zero-shot	basic	0	10.63 (±2.65)	13.95 (±3.11)	12.06 (±2.88)	9.85 (±3.35)	12.16 (±3.81)	8.76 (±3.58)
			role	0	12.17 (±2.81)	10.21 (±2.98)	11.10 (±2.91)	11.05 (±3.51)	9.86 (±3.68)	7.21 (±3.61)
			scene	0	10.33 (±2.77)	11.83 (±3.25)	11.03 (±3.01)	9.79 (±3.47)	10.02 (±3.95)	7.55 (±3.71)
			hybrid	0	10.59 (±2.71)	11.83 (±3.11)	11.18 (±2.85)	9.96 (±3.41)	10.62 (±3.81)	6.48 (±3.55)
		Few-shot	background + hybrid	0	20.16 (±2.51)	12.20 (±2.75)	15.21 (±2.62)	18.14 (±3.21)	11.96 (±3.45)	8.36 (±3.32)
			background + basic	0	25.84 (±2.35)	11.46 (±2.61)	15.88 (±2.48)	23.31 (±3.05)	10.48 (±3.31)	9.60 (±3.18)
			${\mathrm{ref}}_{2}$ + hybrid	24	16.76 (±2.68)	19.68 (±2.99)	18.10 (±2.81)	14.37 (±3.38)	18.01 (±3.69)	10.14 (±3.51)
			${\mathrm{ref}}_{2}$ + basic	24	17.61 (±2.61)	14.69 (±2.88)	16.02 (±2.75)	15.14 (±3.31)	8.87 (±3.58)	6.09 (±3.45)
		Fine-tuning	scene	2725	72.03 (±0.78)	71.86 (±0.85)	71.95 (±0.65)	63.81 (±0.95)	68.55 (±1.05)	64.98 (±0.85)
			role	2725	69.88 (±0.95)	70.49 (±1.01)	70.18 (±0.98)	61.05 (±1.15)	66.92 (±1.21)	62.73 (±1.18)
			hybrid	2725	71.94 (±0.75)	72.48 (±0.81)	72.21 (±0.68)	64.22 (±0.95)	69.01 (±1.01)	65.53 (±0.88)
	Chinese-Alpaca-2-7B	Fine-tuning	scene	2725	72.61 (±0.61)	71.07 (±0.88)	71.83 (±0.55)	63.10 (±0.81)	68.03 (±1.08)	64.51 (±0.75)
			role	2725	73.60 (±0.45)	72.32 (±0.71)	72.96 (±0.38)	65.48 (±0.65)	70.02 (±0.91)	66.89 (±0.58)
			hybrid	2725	72.02 (±0.58)	69.95 (±0.95)	70.97 (±0.49)	62.83 (±0.78)	67.59 (±1.15)	63.81 (±0.69)
	Chinese-LLaMA-2-7B	Fine-tuning	scene	2725	73.56 (±0.51)	71.45 (±0.81)	72.49 (±0.43)	64.91 (±0.71)	69.46 (±1.01)	66.04 (±0.63)
			role	2725	72.82 (±0.65)	68.83 (±1.11)	70.77 (±0.58)	61.33 (±0.85)	68.50 (±1.31)	63.75 (±0.78)
			hybrid	2725	73.46 (±0.48)	70.07 (±0.99)	71.73 (±0.51)	63.08 (±0.68)	69.72 (±1.19)	65.29 (±0.71)
	LLaMA2-Chinese- 7b-Chat	Fine-tuning	scene	2725	66.22 (±0.88)	61.85 (±1.21)	63.96 (±1.05)	56.19 (±1.08)	59.84 (±1.41)	57.23 (±1.25)
			role	2725	68.59 (±0.75)	65.09 (±1.01)	66.80 (±0.81)	58.75 (±0.95)	64.01 (±1.21)	60.18 (±1.01)
			hybrid	2725	69.44 (±0.69)	64.59 (±1.05)	66.93 (±0.79)	59.52 (±0.89)	63.88 (±1.25)	60.95 (±0.99)
	GPT-4	Zero-shot	basic	0	17.13 (±2.02)	59.65 (±2.25)	26.61 (±2.11)	16.43 (±2.52)	58.79 (±2.75)	22.95 (±2.61)
			role	0	17.30 (±1.98)	57.53 (±2.15)	26.61 (±2.05)	16.62 (±2.48)	56.51 (±2.65)	22.45 (±2.55)
			scene	0	18.29 (±2.18)	42.71 (±2.58)	25.62 (±2.35)	17.25 (±2.68)	41.69 (±3.08)	21.73 (±2.85)
			hybrid	0	17.41 (±2.01)	56.41 (±2.21)	26.61 (±2.09)	16.93 (±2.51)	55.72 (±2.71)	20.74 (±2.59)
		Few-shot	background + hybrid	0	30.68 (±1.15)	53.67 (±1.25)	39.04 (±1.18)	28.97 (±1.55)	50.16 (±1.65)	27.35 (±1.58)
			background + basic	0	31.38 (±1.11)	56.66 (±1.19)	40.39 (±1.13)	28.10 (±1.51)	55.14 (±1.59)	28.58 (±1.53)
			${\mathrm{ref}}_{2}$ + hybrid	24	34.62 (±1.05)	40.35 (±1.25)	37.26 (±1.15)	23.37 (±1.45)	37.45 (±1.65)	22.78 (±1.55)
			${\mathrm{ref}}_{2}$ + basic	24	37.56 (±0.98)	40.97 (±1.21)	39.19 (±1.09)	25.59 (±1.38)	43.23 (±1.61)	27.14 (±1.49)
			background + ${\mathrm{ref}}_{2}$ + hybrid	24	26.25 (±1.55)	43.84 (±1.81)	32.84 (±1.68)	20.60 (±2.05)	42.87 (±2.31)	22.04 (±2.18)
			background + ${\mathrm{ref}}_{2}$ + basic	24	37.56 (±1.01)	40.97 (±1.25)	39.19 (±1.11)	27.93 (±1.41)	36.12 (±1.65)	23.65 (±1.51)
			${\mathrm{ref}}_{5}$ + hybrid	60	26.37 (±1.48)	47.82 (±1.65)	34.00 (±1.55)	20.86 (±1.98)	48.03 (±2.15)	23.70 (±2.05)
			${\mathrm{ref}}_{5}$ + basic	60	29.77 (±1.35)	53.80 (±1.51)	38.33 (±1.41)	22.42 (±1.85)	53.05 (±2.01)	26.04 (±1.91)

## Data Availability

The dataset generated and analyzed in this study is publicly available at https://github.com/HongzhiQ/SupervisedVsLLM-EfficacyEval (accessed on 28 May 2024). It includes annotated Weibo posts and evaluation code used for comparing supervised methods and large language models in efficacy evaluation tasks.
